# ﻿Amphibian survey of Ko Pha-gnan in Surat Thani Province, Thailand

**DOI:** 10.3897/zookeys.1207.116758

**Published:** 2024-07-19

**Authors:** Dawn R. Cook-Price, Sunchai Makchai, Sasitorn Hasin, Pongthep Suwanwaree

**Affiliations:** 1 School of Biology, Institute of Science, Suranaree University of Technology, Nakhon Ratchasima, 30000, Thailand Suranaree University of Technology Nakhon Ratchasima Thailand; 2 Thailand Natural History Museum, National Science Museum, Pathum Thani, 12120, Thailand Thailand Natural History Museum, National Science Museum Pathum Thani Thailand; 3 Innovation of Environmental Management, College of Innovative Management, Valaya Alongkorn Rajabhat University under the Royal Patronage, Klong Luang, Pathum Thani, 13180, Thailand Valaya Alongkorn Rajabhat University under the Royal Patronage Pathum Thani Thailand

**Keywords:** Biodiversity, conservation, insular populations, island biogeography, species list

## Abstract

Insular amphibian species are often overlooked, rendering them vulnerable to habitat encroachment and other anthropogenic threats. The aim of this study was to compile a comprehensive list of amphibian species on Ko Pha-ngan in Surat Thani Province, Thailand. Data were collected via transect surveys and drift line fence traps in three different habitat types from February 2021 to September 2023. Our efforts detected 12 unique amphibian species in each of the three habitat types. The highest number of detections was observed in the Ko Pha-ngan-Than Sadet National Park protected areas. The common tree frog (*Polypedatesleucomystax*) and the common Asian toad (*Duttaphrynusmelanostictus*) were the two most abundantly found species on the island, whereas the Koh Tao caecilian (*Ichthyophiskohtaoensis*) and the newly described false Doria’s fanged frog (*Limnonectespseudodoriae*) where the least commonly found species. In addition, *Microhylaheymonsi* and *Fejervaryalimnocharis* tadpoles were observed developing in high-salinity water bodies. Many species have shown a high tolerance in human-dominated landscapes. This study sheds light on the need for additional monitoring to better understand the dynamics of endemic species in addition to the impact tourism-driven development and habitat destruction has on a species with an insularly finite habitat.

## ﻿Introduction

Amphibians, known for their high sensitivity to environmental changes, play a vital role in ecosystems around the world (Liu et al. 2021). Alarmingly, there has been a global decline in amphibian populations, largely attributed to habitat destruction and fragmentation (Hayes et al. 2010). While the significance of biodiversity data concerns a wide range of research areas, its importance becomes paramount when monitoring declining populations for conservation efforts (National Research Council 1992). This worldwide decline in amphibians is mirrored in Thailand, a country rich in amphibian diversity (Chuaynkern and Duengkae 2014).

Ko Pha-ngan situated in Surat Thani Province in southern Thailand, is one of a trio of islands alongside Ko Samui and Ko Tao. Ko Pha-ngan had a historical link as part of the mainland during the Holocene epoch, as part of the Sunda Shelf approximately 21,000 years ago (Nutalaya et al. 1979). This might indicate that it was once part of a more diverse ecosystem. Over time, insularization and subsequent separation from the mainland could have gradually eroded this diversity due to limited habitats and exposure to disturbances. Historically, the island was mined for tin along with a significant portion utilized for coconut plantations (Nutalaya et al. 1979), and currently it is a popular tourist destination (Kaewcharoen et al. 2019). Despite being renowned for its bustling party atmosphere, attracting a large fraction of Thailand’s tourists, Ko Pha-ngan faces significant environmental challenges. In 2017, the island hosted approximately 1.1 million tourists, accounting for 75% of its total visitors (Kaewcharoen et al. 2019). This surge in tourism, a primary economic driver, has inadvertently propelled development and deforestation on the island. Although it sits approximately 80 km away from the mainland, the biodiversity of Ko Pha-ngan, predominantly shielded by the Ko Pha-ngan-Than Sadet National Park, is both unique and understudied (Department of National Parks, Wildlife and Plant Conservation 2018).

Thailand houses over 170 amphibian species and yet the island amphibians remain enigmatic, with considerable gaps in understanding their ecological significance. This knowledge void is particularly noticeable for Ko Pha-ngan, where the herpetofauna remains unstudied. Most islands in Thailand are understudied, and this island offers a unique opportunity for comparison with the few islands that have been studied in Thailand, such as the pristine Tarutao (Nidup et al. 2013), Phi Phi islands (Milto 2014), and the tourist mecca Phuket (Leong et al. 2003). Those islands are in the Andaman Sea on the opposite side of the peninsula, whereas Ko Pha-ngan is in the Gulf of Thailand. This also allows us to look at the effects that humans have on amphibian habitat.

The infrequency of inventory updates from national parks exacerbates this knowledge gap. This study seeks to bridge this chasm by presenting a detailed amphibian species inventory of Ko Pha-ngan. It is our aspiration that this inventory will catalyze more nuanced conservation efforts on the island, ensuring the survival and thriving of its amphibian inhabitants.

## ﻿Methods

Situated in the Gulf of Thailand on the east coast of peninsular Thailand, Ko Pha-ngan is 125 km^2^ (15 km north to south and 10 km east to west), with the Than Sadet-Ko Pha-ngan National Park occupying a third of the island at 42.9 km^2^ and a maximum elevation of 635 m for the entire island (Department of National Parks, Wildlife and Plant Conservation 2018). The island’s lower elevation areas are predominantly residential and agricultural, with montane forests in elevated regions (Koh Phangan City 2023).

**Figure 1.**
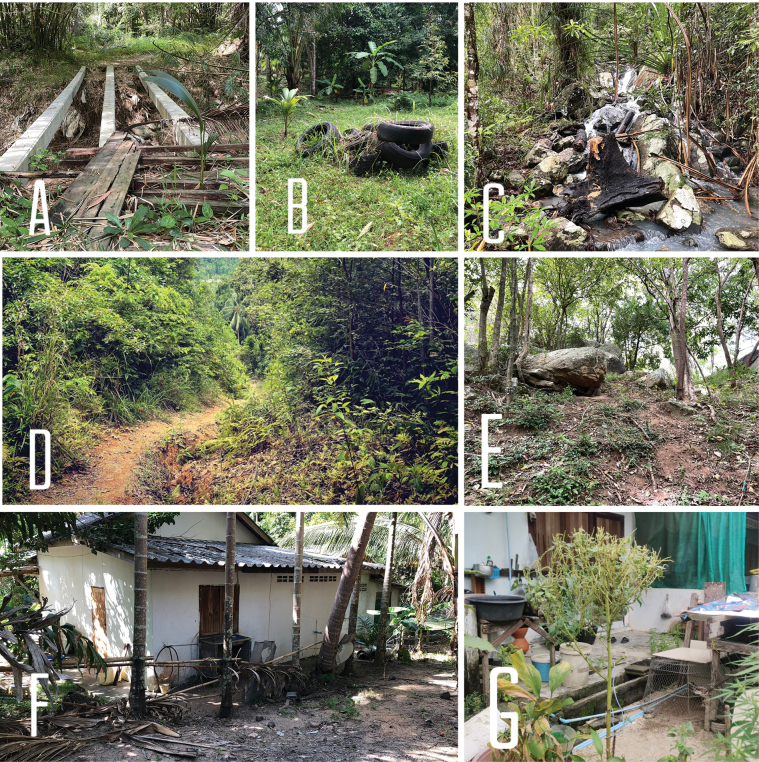
Habitat types **A, B** human-disturbed forest patch **C** rocky river area inside the national park forested area **D** national park forest area **E** human-disturbed forest area **F, G** human-settlement areas. All photos are where species have been found.

**Figure 2.**
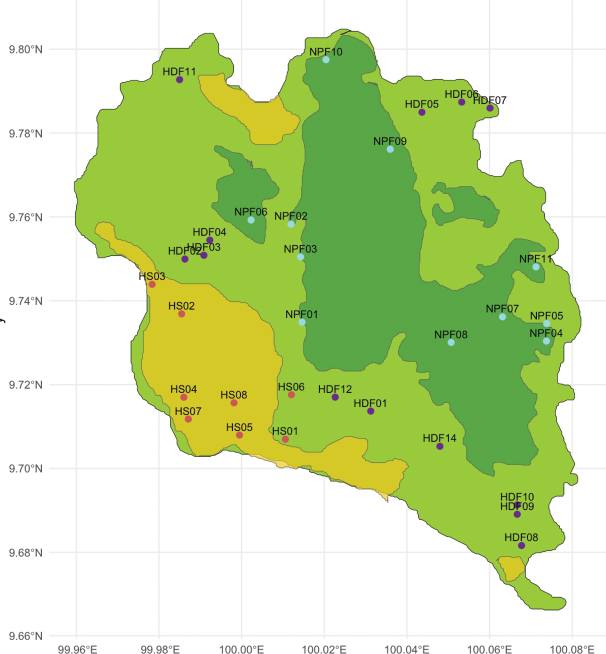
Habitat and transect location map of the island with human settlement (HS) indicated in yellow with red transect points, human-disturbed forest (HDF) areas indicated in light green with purple transect points, and national park forest (NPF) areas indicated in dark green with blue transect points.

Surveys were conducted on the island of Pha-ngan between February 2021 and September 2023. The surveys took place twice per week for a total of 78 weeks under a variety of weather conditions and were carried out between the hours of 19:00 and 02:00 for a total of 1,343 hours. This observational study sought out areas with high detectability potential and remote, less traveled regions. The selection of transects was determined by multiple factors including proximity to water, access to private land, and the safety of the terrain and habitat type. Three major habitats were surveyed (Fig. 1): human settlement (HS), human-disturbed forest (HDF), and National Park Forest (NPF). Human-disturbed forest is any patch of fragmented forest area near human settlements or witnessing human activity in sections throughout. There were 32 transects utilized on the island spanning all three of the different habitats (Figs 1, 2). This includes seven in HS, 13 in HDF, and 11 in NPF areas. Transect lengths varied between 500 m and 1.5 km with variation in elevation from sea level to 630 m. Over half the island consists of montane forested areas, and most of the flat areas are used for residential and agriculture purposes (Koh Phangan City 2023).

Salinity levels were measured with an EZ-9909A multi-functional meter (Yieryi, China) when frogs or tadpoles were detected in water bodies within 100 m of beach-front areas.

**Figure 3.**
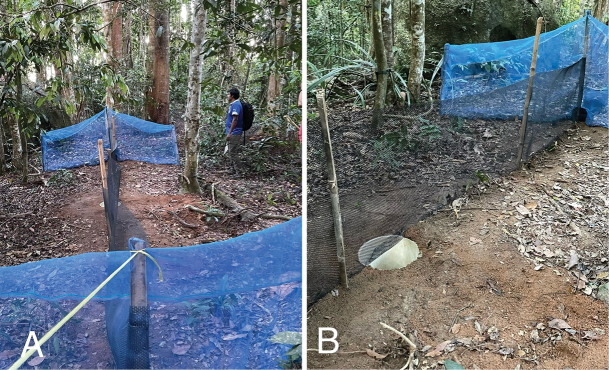
**A** complete view of drift line fence **B** funnel trap used in conjunction with drift line fence.

In addition to foot surveys, seven drift-line fence traps were placed strategically across the island. Configured with a single funnel at one end and a double funnel at the other, the trap also incorporated a pitfall trap in the center (Fig. 3). Configurations were adapted to suit terrain and the length of the funnel trap relative to the available area. The traps, with a 10 m long drift line, offered an alternative means of detection for elusive or reclusive species.

Traps were positioned in the national park forest, human-disturbed forest, and human settlement. They were operational later than foot surveys started as they were open from February 2022 to August 2023, and checked daily for a total of 236 days. A specific trap was open from 7 to 90 days. Each amphibian found was identified in the field when possible before immediate release. The Amphibians of Thailand (Niyomwan et al. 2019) and Thailand’s Natural History Museum database (http://nhmsearch.nsm.or.th) were consulted for identification of the amphibians found. To assess species diversity, we employed two ecological indices: the Shannon–Wiener [H′ = −∑(pi × ln(pi))] and Simpson’s [D=1−∑(pi2]) (Krebs 1989).

## ﻿Results

Our study documents a total of 12 amphibian species on Ko Pha-ngan, which includes 11 species of anurans from 10 genera and one species of Gymnophiona (Table 1). This study expands the Department of National Park’s known records (Department of National Parks, Wildlife and Plant Conservation 2018) by adding five additional anuran species: *Microhylamukhlesuri*, *Occidozygamartensii*, *Limnonectespseudodoriae*, *Hoplobatrachuschinensis*, and *Ichthyophiskohtaoensis*. Of the species surveyed, there are no species listed as a concern on the International Union for Conservation of Nature and Natural Resources (IUCN) list (IUCN 2024).

**Table 1.** Species list and conservation status.

Order	Family	Species	IUCN status
Anura	Microhylidae	*Kaloulapulchra*	LC
		*Microhylaheymonsi*	LC
		Microhylamukhlesuri*	LC
	Dicroglossidae	Occidozygamartensii*	LC
		*Limnonectesblythii*	LC
		Limnonectespseudodoriae*	LC
		*Fejervaryalimnocharis*	LC
		Hoplobatrachuschinensis*	LC
	Ranidae	*Hylaranaerythraea*	LC
	Rhacophoridae	*Polypedatesleucomystax*	LC
	Bufonidae	*Duttaphrynusmelanostictus*	LC
Gymnophiona	Ichthyophiidae	Ichthyophiskohtaoensis*	DD

DD = Data Deficient, LC = Least Concern. *New record from the Than Sadet - Ko Pha-ngan National Park checklist.

*Duttaphrynusmelanostictus* and *Polypedatesleucomystax* were the two most found anuran species on the island, and they were found across all habitats (Table 2). *Duttaphrynusmelanostictus* (common Asian toad) was found more frequently in the National Park Forest habitats along the edges of dirt trails whereas the common tree frog was found more evenly through all habitats. *Ichthyophiskohtaoensis* was the least found anuran, likely due to them being fossorial and nocturnal. The newly discovered *Limnonectespseudodoriae* was found only near or in rocky stream bed areas as described by Yodthong et al. (2021). Though it was found in each habitat, the microhabitat is specialized for this frog as rocky stream areas are the only places they were found. In addition, *Microhylaheymonsi* and *Fejervaryalimnocharis* tadpoles were observed in saline water bodies with measured levels of 3–12 parts per thousand (standard saline levels are 10–35 parts per thousand). Due to the island being surrounded by sea water, there are several water bodies that have high saline levels.

**Table 2.** Amphibian detection by habitat and diversity indices.

	Human settlement	Human-disturbed forest	National park forest	Total
**Diversity Index**				
Shannon-Wiener	2.15	2.17	2.20	
Simpson’s	0.864	0.870	0.870	
**Species**				
*Kaloulapulchra*	103	158	212	473
*Microhylaheymonsi*	72	69	109	250
*Microhylamukhlesuri*	14	18	15	47
*Occidozygamartensii*	88	117	107	312
*Limnonectesblythii*	48	108	102	258
*Limnonectespseudodoriae*	7	45	6	58
*Fejervaryalimnocharis*	39	49	63	151
*Hoplobatrachuschinensis*	15	16	10	41
*Hylaranaerythraea*	108	136	87	331
*Polypedatesleucomystax*	181	175	179	535
*Duttaphrynusmelanostictus*	167	265	370	802
*Ichthyophiskohtaoensis*	15	5	18	38
Total	857	1,161	1,278	3,296

The low number of *Ichthyophiskohtaoensis* (caecilians) detected can be attributed to their nocturnal and fossorial lifestyle, making them less likely to be encountered unless conditions are optimal, such as post-rainfall events. This highlights the importance of survey timing and methodology in detecting species with cryptic behaviors. *Hoplobatrachuschinensis* and *Occidozygamartensii* were detected slightly more in human settlement and human-disturbed forest than in the national park forest, possibly due to their preference and tolerance or vernal cow ponds and muddy marshy fields which are not habitats often found in the national park forest.

For diversity analysis, the NPF area has the highest Shannon–Wiener value with HDF and HS closely following in values (Table 2). The Simpson’s Index revealed the same values for both HDF and NPF, and only slightly different for HS. While the differences among these habitats were not statistically significant, the slightly higher diversity indices in NPF regions suggest a slightly more varied amphibian population.

These indices offer complementary insights; Shannon–Wiener places greater emphasis on species richness and evenness. A higher value indicates a more diverse community, where species are not only numerous but also more evenly distributed. The Simpson’s Index focuses more on the dominance of a particular species. A lower value of a Simpson’s Index indicates a higher diversity, meaning that the ecosystem is not dominated by one or a few species but has a more balanced distribution of species. This dual approach allows for a more nuanced understanding of amphibian diversity across different habitats. Though the national park forest habitat had slightly more diversity than the other habitats, the frogs that adapted to human settlement and human-disturbed forests seem to thrive which is consistent with these common species throughout Thailand.

Notably, the national park forest area, while slightly higher in diversity indices, did not differ significantly from human-disturbed forest or human settlement, indicating that amphibian populations are relatively similar across these environments. This similarity is remarkable, considering the varying degrees of human impact, and implies that species such as *Kaloulapulchra* and *Microhylaheymonsi*, which are abundant across all habitats, are the resilient and adaptable to changes in their ecosystems. This indicates a healthier, more balanced amphibian community and a flexibility or resilience among the common species in Thailand.

### ﻿Taxonomy

**﻿Class Amphibia**

**Order Anura**

#### Family Microhylidae Günther, 1858

##### ﻿ Kaloula pulchra

Taxon classificationAnimaliaAnuraMicrohylidae

Gray, 1831

B1059FA1-BB13-5620-83C0-EA040CA74FD1

**Figure 4.**
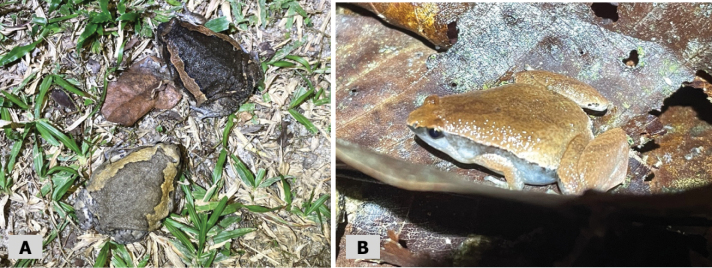
**A** two color variations of *Kaloulapulchra*, observed in human settlement **B***Microhylaheymonsi* observed in leaflitter.

Fig. 4 Banded bullfrog, painted bullfrog

###### Notes.

These fossorial individuals were occasionally observed in tree holes (7), burrows (4), termite mounds (3) and anthropogenic material (3). Once observed in a dirt track puddle of saline water.

###### Distribution.

This species is abundant throughout the island and found in all habitat types. Individuals were observed in all human-settlement transect areas which includes garden areas near homes, fallow fields between human development, edge habitat near human-disturbed forest. This frog was detected in all human-disturbed forest including patches between developed housing areas. Frogs were detected in all national park forest transects. More often detected near water.

##### ﻿ Microhyla heymonsi

Taxon classificationAnimaliaAnuraMicrohylidae

Vogt, 1911

7D43CC51-83A4-598A-BD5F-74C025ACFA80

Fig. 4 Dark-sided chorus frog

###### Notes.

A total of 250 individuals were observed in a variety of habitat, including near ponds, puddles, grassy areas, streams, and house or garden areas in and around water pots common in garden areas. Found in primarily around freshwater; however, four individuals were also observed in and around brackish and saline water located in human settlement.

###### Distribution.

This species was detected at three of the seven human-settlement areas, six of the 14 human-disturbed forest habitats, and six of the 10 national park forest habitats.

##### ﻿ Microhyla mukhlesuri

Taxon classificationAnimaliaAnuraMicrohylidae

Hasan et al., 2014

14BE3732-194D-56DB-B91C-009E4A70F26E

**Figure 5.**
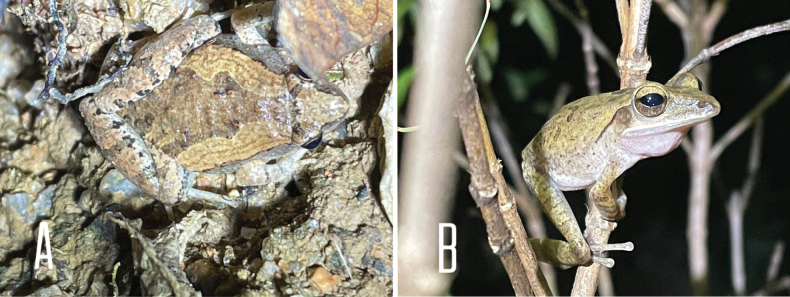
**A***Microhylamukhlesuri* near a small pond in human-disturbed forest **B***Polypedatesleucomystax* found on low branches near small pond in a marsh area in human-disturbed forest.

Fig. 5 Mukhlesur’s narrow-mouthed frog

###### Notes.

Individuals were observed in a variety of habitats. Three individuals were found in a rocky stream bed in national park forest. Eighteen individuals were observed on sandy trails at the edge of both forested and scrub-grassy habitats, five individuals were found in a patch of human-disturbed forest at the edge of human settlement, seven individuals observed in a grassy area in human settlement on the edge of a human-disturbed forest, five individuals observed in a grassy area at the edge of a pond in human settlement, and nine individuals were found in the leaf litter near a pond at the edge of human-disturbed forest. Primarily found around freshwater; however, it was also observed in and around brackish and saline water. Tadpoles observed developing in saline water (August 2023).

###### Distribution.

This species was detected in three of the eight human-settlement transects, three of the 13 human-disturbed forest transects, and seven of the 11 national park forest transects.

#### Family Rhacophoridae Hoffman, 1932 (1858)

##### ﻿ Polypedates leucomystax

Taxon classificationAnimaliaAnuraRhacophoridae

(Gravenhorst, 1829)

7FEFECE6-AEC8-559A-B39B-E0AB4A2C7CB0

Fig. 5 Southern Clade. Common tree frog or four-lined tree frog

###### Notes.

This species was the second most observed on the island; 535 individuals were commonly observed throughout the island in a variety of habitats including forested areas near water sources such as streams or ponds, grassy plantation areas near water sources, such as overgrown vegetation patches near ponds, disturbed forested areas, and human habitat near streams, ponds, or anthropogenic structures holding water. Observed once in a concrete basin of a water fountain in a human-settlement area at the edge of a stream.

###### Distribution.

The common tree frog is distributed throughout peninsular Thailand south of the Isthmus of Kra and is part of the southern clade of this species group (Buddhachat and Suwannapoom 2018). This species is common throughout the island with detection in all 8 of human-settlement transects, 12 of the 13 human-disturbed forest transects, and 10 of the 11 national park forest transects.

#### Family Ranidae Rafinesque, 1814

##### ﻿ Hylarana erythraea

Taxon classificationAnimaliaAnuraRanidae

(Schlegel, 1837)

7A3BFC4C-BDB8-5596-8515-2C4FF1AE6BFC

**Figure 6.**
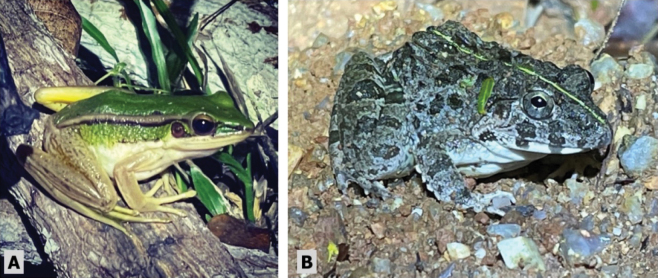
**A***Hylaranaerythraea* found on a fallen tree branch in a human-disturbed forest patch adjacent to human settlement **B***Fejervaryalimnocharis* on dirt trail between a stream and grassy area at in national park forest area.

Fig. 6 Green grass frog

###### Notes.

The 331 individuals detected were commonly observed near ponds and some stream areas. Observations were made on the ground in grass or sandy soil, in water of a pond, on fallen trees, and in low areas on the side of trees. This species was observed in or near fresh, brackish, and saline water.

###### Distribution.

The green grass frog is distributed throughout the island, most commonly near water. Detection occurred in five of the eight human-settlement transects, 13 of the 13 human-disturbed forest transects, and five of the 11 national park forest transects.

#### Family Dicroglossidae Anderson, 1871

##### ﻿ Fejervarya limnocharis

Taxon classificationAnimaliaAnuraDicroglossidae

(Gravenhorst, 1829)

80EF4EBC-E447-57A9-93D5-79EB039A2B0F

Fig. 6 Pond frog

###### Notes.

We observed 327 individuals near ponds and other water bodies such as puddles or still water near stream areas. Found in fresh, brackish, and saline water habitat. Tadpoles observed through to froglet in saline water puddle.

###### Distribution.

This species is common throughout the island with detection in six of the eight human-settlement transects, eight of the 13 human-disturbed forest transects, and six of the 11 national park forest transects.

##### ﻿ Phrynoglossus martensii

Taxon classificationAnimaliaAnuraDicroglossidae

Peters, 1867

967F470D-F2DD-5ACB-A4E5-46DEF8CBB4A1

Fig. 7 Puddle frog

###### Notes.

We observed 151 individuals in puddles situated in dirt track paths in human-disturbed forest and human-settlement areas such as a cement fountain not in use but still retaining water.

**Figure 7.**
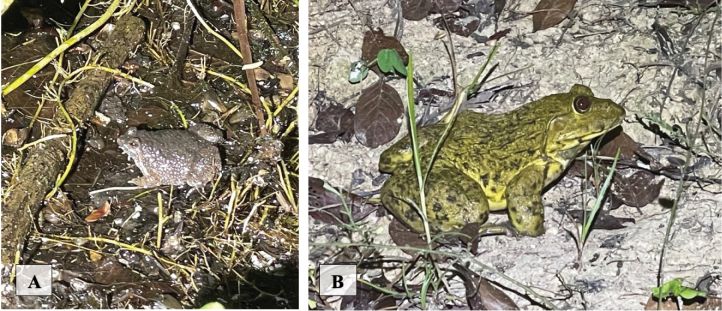
**A***Phrynoglossusmartensii* found in a water fountain at an abandoned party location in human-disturbed forest **B***Hoplobatrachusrugulosus* at the edge of a small pond in a human-disturbed forest patch in human settlement.

###### Distribution.

This species is distributed throughout Thailand but is more prevalent in central and peninsular areas (Köhler et al. 2021). It is common throughout the island near small water bodies such as puddles and small vernal ponds. Individuals were detected in seven of the eight human-settlement transects, 11 of the 13 transects in human-disturbed forest areas, and six of the 11 transects in national park forest areas.

##### ﻿ Hoplobatrachus chinensis

Taxon classificationAnimaliaAnuraDicroglossidae

(Wiegmann, 1834)

2DEC8D2A-6B40-56A2-8E35-765FD6C5DF68

Fig. 7 Chinese edible frog

###### Notes.

Individuals were sometimes found in or on the edge of small ponds or vernal water holes used by water buffalo in marshy fallow fields. It is often found in fresh food markets and has been farmed on the island.

###### Distribution.

The species was found near water bodies such as small ponds or standing water areas sporadically throughout the island. Individuals were observed in two of the eight areas in human habitat, two of the 13 areas in human-disturbed forest, and only two of the 11 areas surveyed in national park forest.

##### ﻿ Limnonectes blythii

Taxon classificationAnimaliaAnuraDicroglossidae

(Boulenger, 1920)

9A136997-2D0D-5354-8454-5C2CFCAA4EF3

Fig. 8 River frog

###### Notes.

**Figure 8.**
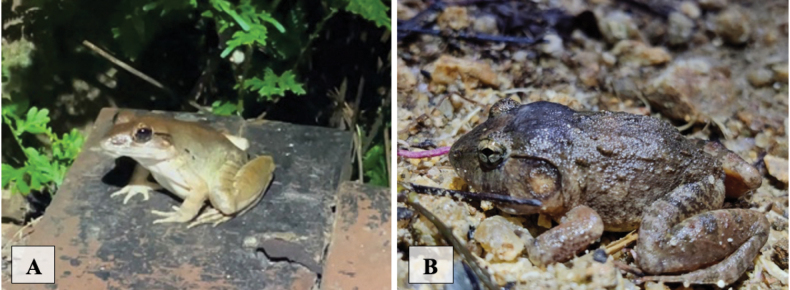
**A***Limnonectesblythii* found on a bridge over a river near human settlement and a small, disturbed forest patch **B***Limnonectespseudodoriae* found on a rocky dirt path parallel to a rocky stream wash.

The 273 individuals detected were primarily observed on the banks of rivers or edges of ponds and rocky riverbeds. The majority (225) were observed in both national park forest and human-disturbed forest areas. The individuals found in human-settlement areas were adjacent or within 25 m of a stream or marshy water source. Fig. 8 shows an individual on a bridge walkway above a stream on dormant party grounds inhabited by villagers.

###### Distribution.

The river frog is distributed throughout the island in or near stream or pond areas with individuals observed in five of the eight human-settlement transects, 10 of the 13 human-disturbed forest transects, and eight of the 11 in national park forest transects.

##### ﻿ Limnonectes pseudodoriae

Taxon classificationAnimaliaAnuraDicroglossidae

(Yodthong, Rujirawan, Stuart & Aowphol, 2021)

ADE9F36B-1ED6-5840-B829-95BD6FEE7856

Fig. 8 False Doria’s fanged frog

###### Notes.

This species was observed in or around rocky stream habitat. Eggs were observed on land at the edge of a pool of standing water at a leveled area of a rocky stream wash in national park forest. This newly described species has been documented on only three islands, Ko Pha-ngan, Ko Samui, and Ko Lanta (Yodthong et al. 2021). The individuals observed in the human-settlement area were in a flooded stream area between a house and small pond.

###### Distribution.

This species was detected primarily in rocky river systems. Individuals were detected in one of the eight human-settlement transects, three of the 13 human-disturbed forest transects and five of the 11 national park forest transects.

#### Family Bufonidae Gray, 1825

##### ﻿ Duttaphrynus melanostictus

Taxon classificationAnimaliaAnuraBufonidae

(Schneider, 1799)

6C637E27-01D5-5CA5-A731-C640B602232F

**Figure 9.**
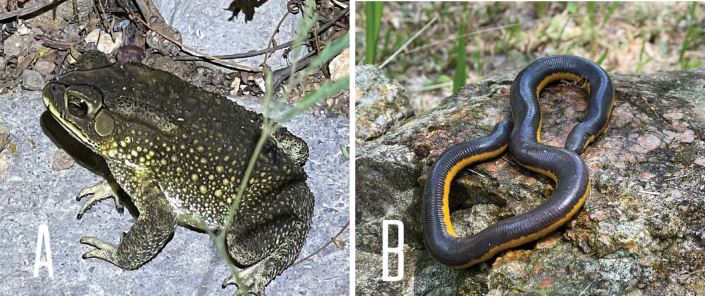
**A***Duttaphrynusmelanostictus* found on cement patch on a dirt track in humansettlement area in a human-disturbed forest **B***Ichthyophiskohtaoensis* found under an overturned rock in a garden in human habitat not far from a stream.

Fig. 9 Asian common toad

###### Notes.

This species was the most observed species on the island; 559 individuals were commonly observed throughout the island on dirt paths, dirt roads, and pooled bodies of water in forested stream areas.

###### Distribution.

The Asian common toad is distributed throughout the island with detection in all eight of the human-settlement transects, all 13 of the human-disturbed forest transects, and all 11 of the national park forest transects.

#### Family Ichthyophiidae Taylor, 1968

##### ﻿ Ichthyophis kohtaoensis

Taxon classificationAnimaliaAnuraIchthyophiidae

Taylor, 1960

338E74BC-BA49-5544-9DA6-B99C896BCAE1

Fig. 9 Koh Tao caecilian

###### Notes.

Forty individuals were observed near and around marshy habitat, under anthropogenic items near marshy river overflows, and occasionally under rocks in rocky dirt patches near rivers. Most often detected after or during rain, and commonly found in human-settlement areas after digging or gardening.

###### Distribution.

This caecilian is distributed throughout the island, although it is fossorial and not easily detected. Individuals were detected in two of the eight human-settlement transects, five of the 13 human-disturbed forest transects, and three of the 11 national park forest transects.

## ﻿Discussion

In more recent history, Ko Pha-ngan’s landscape has evolved considerably. Formerly a tin mining hub, it transitioned to a mosaic of plantations with scattered remnants of native forests (Nutalaya et al. 1979). The burgeoning tourism sector compounds these changes by encroaching on essential habitats. Such rampant habitat modifications, while economically justifiable, potentially imperil the island’s dwindling biodiversity (Russell and Kueffer 2019), as habitat fragmentation can reduce species diversity (Berger-Tal and Saltz 2019). Such disturbances pose unique challenges for island ecosystems, where specialized species are particularly susceptible (Kanowski et al. 2006).

All the species found are widely distributed across Thailand, demonstrating considerable adaptability to varying habitats. *Polypedatesleucomystax* and *Kaloulapulchra*, for instance, are often spotted near human habitations, whereas *Limnonectesblythii* seems to favor riverine environments, indicating specific habitat preferences. This adaptability is evident in the face of rapid environmental changes, hinting at why some species flourish while others are at risk (Liu et al. 2021). This island’s species composition can be compared to findings from other regions to gain insights into biodiversity patterns and potential influencing factors. A comparison of our findings with studies from other island regions and mainland habitats might give more insight to the understanding of the diversity observed on Ko Pha-ngan. Tarutao, a protected and more pristine island located in the southern peninsular Satun Province approximately 25 km from peninsular mainland (Cocks et al. 2005), houses 10 amphibian species (Nidup et al. 2013), which is less than the number found on Pha-ngan island. The topography of Tarutao differs slightly from Ko Pha-ngan, as that island has limestone cliffs and is relatively untouched and nestled in the Andaman Sea; however, there are similarities such as size (152 km^2^) and elevation (713 m). Four of species on Tarutao, namely *K.pulchra*, *L.blythii*, *P.leucomystax*, and *H.erythraea*, also occur on Ko Pha-ngan. The distinct species on Tarutao suggest regional variations and could be influenced by Tarutao’s specific environmental conditions, land protection, and proximity to other biodiversity hotspots.

Phuket, the largest island in Thailand (543 km^2^), and the nearby islands of Yao Noi (45 km^2^) and Yao Yai (92 km^2^) are much closer to the mainland. Phuket is connected by a bridge less than 1 km long, and the two smaller islands are much less developed than Phuket. Phuket has 26 amphibian species (Leong et al. 2003), while Yao Noi and Yao Yai, nestled between Phuket and the mainland, have 19 species each (Visoot et al. 2023). Eleven species on Ko Pha-ngan are also found on these three islands. The only difference was the caecilian which has not been documented on Yao Noi and Yao Yai. Species such as *Leptobrachiumsmithi* prefer to breed in slow moving river areas or small side pools near riverbeds, and this is not a consistent habitat on Ko Pha-ngan. In addition, species such as *Chalcoranaeschatia* and *Phrynoidisasper* prefer river and riverine habitats. Though Ko Pha-ngan has river habitat, during the dry season many of the river systems dry up, which may account for some of the variation in species present. In addition, these species are found in primary or significant secondary forest areas. Despite the tourism similarity, patches of forest differ between the islands.

On the opposite side of the peninsula in the South China Sea, Bidong Island on Malaysia’s east coast houses only three amphibian species (*K.pulchra*, *P.leucomystax*, and *M.heymonsi*), likely due to the island’s degraded habitat (Fatihah-Syafiq et al. 2020). All these species are found throughout the peninsula, including on Pha-ngan island. The low number of amphibian species can possibly be attributed to areas of the island having not been explored.

On the eastern side of the Gulf of Thailand, the Koh Man Islands are near (7 km) the mainland and only have four anuran species. Three of which are the same as on Ko Pha-ngan (*Fejervaryalimnocharis*, *Kaloulapulchra*, and *Duttaphrynusmelanostictus*), with only one difference, *Fejervaryacancrivora* (Chan-ard and Makchai 2011).

In the Surat Thani province on the mainland, there are at least 38 amphibian species from Khao Sok National Park (Thai National Parks 2023), and this diversity is much higher than on Ko Pha-ngan. Similar to Surat Thani, the Phang-nga province has 39 species (Pauwels et al. 2002); however, Phuket is much closer to its mainland counterpart (Phang-nga) than Ko Pha-ngan is to Surat Thani. A plausible explanation for these variations in biodiversity is the distance from the mainland of these islands. Tarutao and Ko Pha-ngan, approximately 25 km and 80 km from the mainland, hold fewer species than Phuket, which is just 660 m away and linked by a bridge. Yao Noi and Yao Yai, both within 20 km from the mainland, also support this trend. The MacArthur and Wilson (1967) biogeographical theory suggests that species diversity diminishes with increased isolation. A study in the Yoddom Wildlife Sanctuary in northeastern mainland Thailand further bolsters this claim; it reported a diverse amphibian population of 26 species (Thongproh et al. 2019), which contrasting starkly with the island findings.

While some amphibian species (*Polypedatesleucomystax* and *Duttaphrynusmelanostictus*) demonstrate adaptability to Ko Pha-ngan’s shifting environment, others are at risk, particularly those species endemic to specific habitats such as *Limnonectespseudodoriae*. River species, such as *Amolopspanhai* and *Sylviranamalayana*, found on Phuket and Surat Thani mainland were not detected on Ko Pha-ngan, as they are primarily found in areas with rocky, flowing rivers, and that specific habitat is not consistent on Ko Pha-ngan. In addition, Ko Pha-ngan does not have any *Rhacophorus* (gliding frog) species, which are commonly detected in the tree canopy above or near water sources in the Surat Thani and Phang-nga provinces. The comparative lack of biodiversity can be attributed to factors like distance from the mainland and the island’s environmental history. The conspicuous absence of some mainland species might be attributed to historical isolation, compounded by recent human activities, and the limitation of the researchers’ observation.

To augment our understanding, in-depth research in the island’s remote forested locales is pivotal. Prolonged studies might reveal a more nuanced diversity profile. While the Surat Thani mainland has undergone rigorous examination, its islands remain terra incognita for amphibian research.

## Supplementary Material

XML Treatment for Kaloula pulchra

XML Treatment for Microhyla heymonsi

XML Treatment for Microhyla mukhlesuri

XML Treatment for Polypedates leucomystax

XML Treatment for Hylarana erythraea

XML Treatment for Fejervarya limnocharis

XML Treatment for Phrynoglossus martensii

XML Treatment for Hoplobatrachus chinensis

XML Treatment for Limnonectes blythii

XML Treatment for Limnonectes pseudodoriae

XML Treatment for Duttaphrynus melanostictus

XML Treatment for Ichthyophis kohtaoensis

## ﻿Additional information

### Author ORCIDs

Dawn R. Cook-Price https://orcid.org/0009-0000-5035-9642

Sunchai Makchai https://orcid.org/0009-0008-4067-7442

Sasitorn Hasin https://orcid.org/0000-0003-0704-2724

Pongthep Suwanwaree https://orcid.org/0000-0002-3047-2717

### Data availability

All of the data that support the findings of this study are available in the main text.
